# mNGS helped diagnose scrub typhus presenting as a urinary tract infection with high D-dimer levels: a case report

**DOI:** 10.1186/s12879-021-06889-9

**Published:** 2021-12-07

**Authors:** Mei-fang Liu, Yong Liu, De-rong Xu, La-gen Wan, Rui Zhao

**Affiliations:** 1 Department of Clinical Laboratory, The First Affiliated Hospital of Nanchang University, Nanchang, 330006 Jiangxi People’s Republic of China; 2 Department of Emergency, The First Affiliated Hospital of Nanchang University, Nanchang, 330006 Jiangxi People’s Republic of China

**Keywords:** Scrub typhus, Metagenomics next-generation sequencing, *O. tsutsugamushi*, Urinary tract infection

## Abstract

**Background:**

Scrub typhus is caused by *O. tsutsugamushi* and spreads through mite larvae biting the skin. Classic symptoms of the disease are eschar and lymphadenopathy. Previous reports have revealed clinical manifestations of scrub typhus, including gastrointestinal symptoms, meningoencephalitis, ocular flutter, pneumonitis, acute respiratory distress syndrome, and acute kidney injury. However, cases of scrub typhus presenting as a urinary tract infection (UTI) with high D-dimer levels could be easily misdiagnosed when clinical attention is insufficient, resulting in difficulty in making a timely diagnosis of the infection. Metagenomics next-generation sequencing (mNGS) is a revolutionary and highly sensitive method that may help in diagnosing atypical cases, even when trace amounts of pathogens are present.

**Case presentation:**

A 52-year-old female presented with a 10-day history of fever, chills, headache and myalgia. She was initially diagnosed with influenza at a local clinic. Various antibacterials were used on the 2nd–12th day onwards; however, her symptoms persisted and were followed by increased urination duration, frequency, urgency and dysuria for 2 days. *Orientia tsutsugamushi* was confirmed as the pathogen responsible for the infection through mNGS analysis of her blood samples from Day 13 onwards. The patient’s temperature changed remarkably 24 h after the initiation of doxycycline. Over the next 48 h (i.e., Day 15 onwards), the patient showed clinical improvement. She recovered and was discharged from the hospital.

**Conclusions:**

Scrub typhus can present atypical clinical symptoms, such as UTIs, in a febrile patient. mNGS may be a useful method for identifying *O. tsutsugamushi* infection in patients with atypical clinical manifestations.

## Background

Scrub typhus is an acute infectious disease caused by *O. tsutsugamushi,* which is transmitted to humans through the bite of infected mites (chiggers) [1]. In southern China, the epidemic season of the illness is from May to December, with a peak from June to September, and the annual incidence rate in Jiangxi Province is 0.98 per 100,000 [2]. Clinical manifestations of scrub typhus include fever, headache, myalgia, and gastrointestinal symptoms. An eschar is often found at the inoculation site. Lymphadenopathy is found proximally (draining node). In patients with severe cases, the disease can progress to the development of interstitial pneumonitis, acute respiratory distress syndrome, meningoencephalitis, and acute kidney injury [1]. The fatality rates of untreated and treated scrub typhus have been reported to be 6% and 1.4%, respectively [3]. Notably, the mortality rate of patients with multiorgan failure may be as high as 24% [4], which is a fourfold and 17-fold increase compared to those of patients with untreated and treated scrub typhus, respectively. Thus, early diagnosis of scrub typhus is crucial to improve outcomes. The routine diagnostic methods for scrub typhus include serologic tests, nucleic acid amplification tests (NAATs), and tissue biopsies [5]. Serologic tests for *O. tsutsugamushi* generally become positive only after 7–10 days of symptom onset and sometimes even 25 days later, which could easily delay treatment [6]. Nested conventional PCR is prone to amplicon contamination. Moreover, NAATs are more sensitive in cases of acute illness [7]. Tissue biopsies of *O. tsutsugamushi* are challenging and dangerous, and biopsy samples should be handled in biosafety level 3 (BSL-3) laboratories; however, almost all hospital laboratories are BSL-2, which limits the application of this diagnostic method [7]. Metagenomics next-generation sequencing (mNGS), a nontargeted and quick diagnostic method, has been shown to identify new and unknown species of *Rickettsia* carried by vectors or hosts [8] and is therefore especially suitable for the diagnosis of rare, novel, and atypical aetiologies of complicated infectious diseases [9].

More than 80% of urinary tract infections (UTIs) are caused by *Escherichia coli* and rarely by other bacteria, including *Staphylococcus*, *Klebsiella*, *Enterobacter*, *Proteus*, and *Enterococcus* [10]*.* UTIs caused by *O. tsutsugamushi* have rarely been reported. D-dimer, a soluble fibrin degradation product, serves as an available biological indicator of haemostatic abnormalities and intravascular thrombosis and is routinely used to exclude venous thromboembolism and diagnose and monitor disseminated intravascular coagulation (DIC) [11]. We successfully diagnosed a case of atypical scrub typhus presenting as a UTI accompanied by high D-dimer levels using mNGS. The patient was ultimately cured and discharged.

## Case presentation

**Fig. 1:**
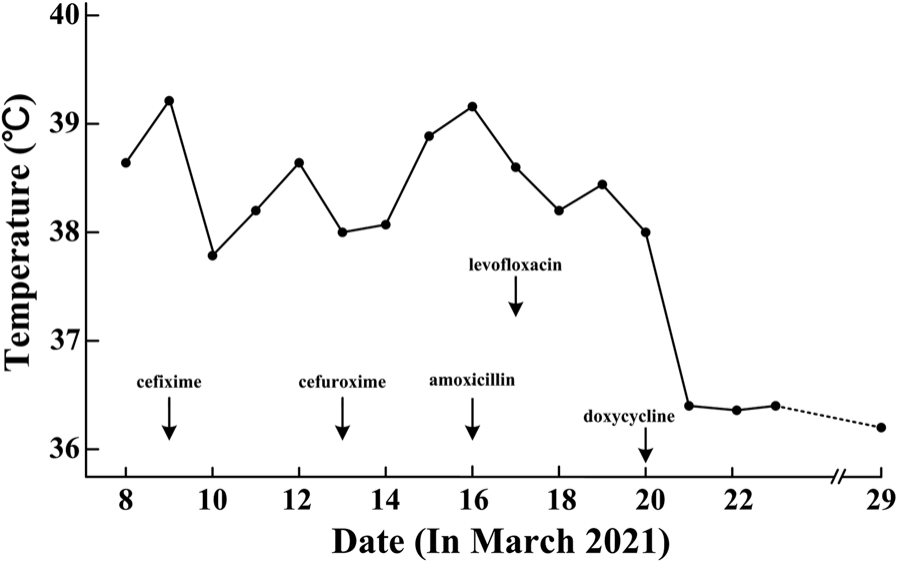
The medication and average daily temperature of the patient from the onset of illness

A 52-year-old woman, an urban dweller with a history of fieldwork, complained of fever, chills, headache, and myalgia for 10 days and dysuria and urinary frequency and urgency for 2 days and was admitted to the First Affiliated Hospital of Nanchang University in March 2021. Before coming to our hospital, the patient had been diagnosed with influenza at a local clinic and subsequently received cefixime for 5 days, cefuroxime for 4 days, and amoxicillin for 1 day (Fig. 1); however, her symptoms remained. On admission, the patient presented with a temperature, pulse rate, respiration rate, blood pressure, and Barthel index of 39.3 °C, 103 beats/min, 25 beats/min, 90/44 mmHg, and 90 points, respectively. There were no rashes or eschar, no lymphadenopathy, and no obvious abnormalities on her body. Laboratory examination showed elevated serum amyloid A protein (SAA) (266.47 mg/L), C-reactive protein (CRP) (200.98 mg/L), alanine aminotransferase (ALT) (61.5 U/L), aspartate aminotransferase (AST) (64.7 U/L), D-dimer (22.84 mg/L), prothrombin time (PT) (14.1 s), and prothrombin time/international normalized ratio (PT-INR) (1.11) values. Abnormalities in proteinuria (1 +), the white blood cell count (WBC) in urine (6–8 cells/high-power field (HPF)) and haematuria (2 +) were noted. The WBC (7.93 × 10^9^/L) and PLT (118 × 10^9^/L) levels were normal. There were no obvious abnormalities in the remaining test results (Table 1). Her body temperature fluctuated at approximately 38 °C after admission to the emergency medicine department (Fig. 1). A febrile infectious disease of unknown etiology was diagnosed at our hospital. Considering that it was likely to be caused by a bacterial or viral infection, levofloxacin and peramivir were used for temporary treatment, and diclofenac sodium was used to treat fever when necessary, but the patient's body temperature dropped only temporarily and then rose again after a few hours. This showed that the treatment was not optimal.

**Table 1:** Results of laboratory tests of patients at different times

Categories	Reference range	First day of hospitalization (2021/3/17)	After 3 days of doxycycline (2021/3/23)	One week after discharge (2021/3/29)
Blood routine examination				
WBC (10^9^/L)	3.5–9.5	7.93	8.19	9.82
Neutrophil (10^9^/L)	1.8–6.3	6.3	3.53	5.02
SAA (mg/L)	0–10	**266.47**	**8.64**	2.21
CRP (mg/L)	0–8	**200.98**	**16.53**	0.6
Urinalysis				
Proteinuria	Negative	**1 + **	Negative	Negative
Urine RBC (cells/HPF)	0–3	**80 cells/L**	3 cells/L	2 cells/L
Urine WBC (cells/HPF)	0–5	6–8	0–2	0–1
Haematuria	Negative	**2 + **	Negative	Negative
Urine culture		No growth	No growth	Nil
Blood coagulation panel				
PT (s)	9.8–12.1	**14.1**	11.1	10.3
PT-INR	0.85–1.15	**1.22**	1.01	0.97
D-dimer (mg/L)	0–0.55	**22.84**	**5.21**	0.22
Liver panel				
ALT (U/L)	7–40	**61.5**	**128.4**	38.2
AST (U/L)	13–35	**64.7**	**125.8**	30.9
Other blood test				
Blood culture		No growth	No growth	Nil

Abnormal values are marked in bold

**Fig. 2:**
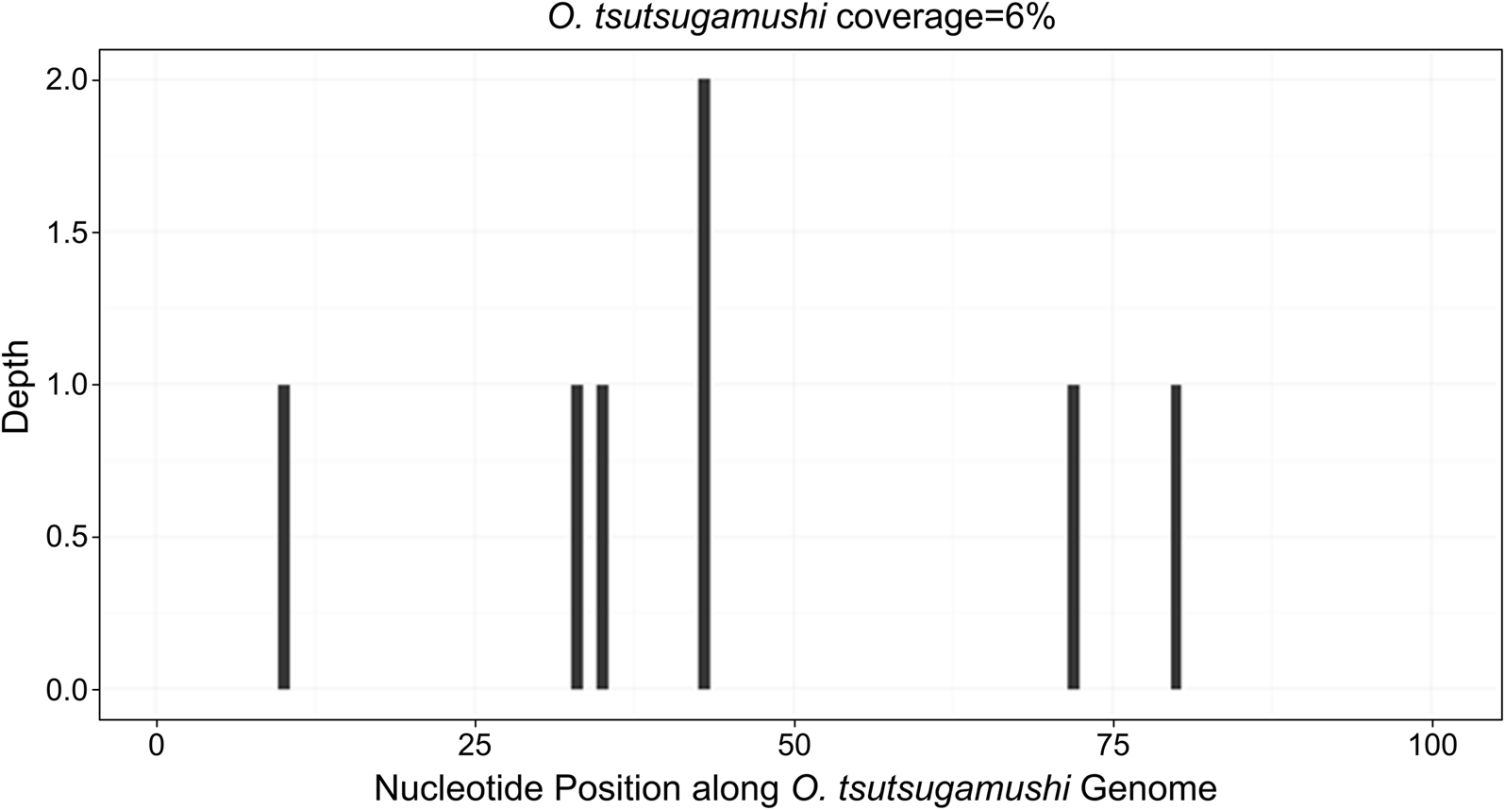
*O. tsutsugamushi* was detected in whole blood samples by metagenomics next generation sequencing. Seven sequence reads were identified, with a coverage rate of 6%

To make a definitive diagnosis, blood samples were collected on Day 11 of illness (18 March) for serologic tests of common bacteria such as typhoidal *Salmonella*, *Streptococcus pneumoniae*, *Mycoplasma pneumoniae* and *Chlamydia pneumoniae*; viruses such as herpes simplex virus, influenza A virus, influenza B virus, rubella virus, adenovirus, respiratory syncytial virus and coxsackie virus; parasites such as *Toxoplasma gondii*; and autoimmune diseases. In addition, blood and urine cultures were performed. The results of these tests were negative. Unfortunately, the patient’s symptoms worsened, and her blood pressure continued to drop (84/41 mmHg). Considering the complexity of the patient’s condition, whole-blood samples were sent to the molecular diagnostic laboratory for mNGS analysis. Two days later, the results showed that *O. tsutsugamushi* was the causative pathogen, and 7 sequence reads were identified, with a coverage rate of 6% (Fig. 2).We substituted levofloxacin and peramivir with doxycycline at a dosage of 100 mg every 12 h. The patient’s body temperature returned to normal after 24 h. The symptoms of UTI slowly resolved, and the D-dimer levels declined. After 3 days of treatment (e.g., 23rd March), laboratory tests showed that the levels of SAA (8.46 mg/L), CRP (16.53 mg/L), and D-dimer (5.21 mg/L); the PT (11.1 s); and the WBC in urine (0–2 cells/HPF) had declined drastically, and proteinuria and haematuria had become negative. The WBC (8.19 × 10^9^/L), PLT (314 × 10^9^/L), and blood pressure (105/65 mmHg) levels were normal, indicating that a therapeutic effect had been achieved. The patient recovered and was discharged. On the 7^th^ d after discharge, all examination results were normal (see Table 1).

## Discussion and conclusions

Scrub typhus, a potentially life-threatening disease with an enormous incidence in the Asia–Pacific Rim, remains remarkably neglected [12]. Outdoor work is the high-risk occupation associated with scrub typhus [13]. The typical clinical manifestation of scrub typhus is an eschar at the site of mite feeding. Depending on the geographic area and research area, an eschar is found in 1% and 97% of patients, respectively, and is generally considered a key clinical feature [14]. Patients without an eschar are difficult to diagnose. The current patient, an urban dweller with a history of field work, was previously in good health. Unfortunately, the patient was not diagnosed in a timely manner because scrub typhus was not among the differential diagnoses considered by the clinicians given the rarity of scrub typhus in our region and the lack of typical clinical features; therefore, tests such as specific serologies and NAATs were not ordered. Additionally, her main symptoms were urinary, which are rarely reported in cases of scrub typhus. Only Shu et al. [15] and Bhattarai et al. [16] have reported UTIs associated with scrub typhus. The mechanism of urinary involvement in scrub typhus may be that *O. tsutsugamushi* induces vasculitis [17]. High D-dimer levels may occur because *O. tsutsugamushi* mainly attacks endothelial cells, which have procoagulant and proinflammatory properties [18]. The coagulation system is activated, and the resulting thrombin converts soluble fibrinogen to fibrin monomer, leading to increase in D-dimer levels [11]. The laboratory diagnosis of scrub typhus includes serologic tests, NAATs, and tissue biopsies. Indirect immunofluorescence assay (IFA) requires the availability of fluorescence microscopes and professional testing personnel, neither of which is usually available in endemic areas [19]. The Weil–Felix agglutination test has poor sensitivity and specificity due to the lack of species identification techniques. Moreover, cross-reactions between *O. tsutsugamushi* and other pathogens, such as those associated with dengue, malaria, typhoid, influenza and leptospirosis, should be noted [19]. Real-time polymerase chain reaction (Q-PCR) presents 97% sensitivity and 100% specificity for the diagnosis of scrub typhus [19]. However, laboratories in many countries, including China, have not routinely performed this diagnostic test, and PCR can be applied when an eschar is present. However, it is difficult to suspect and determine in advance a diagnosis of scrub typhus if there are no typical clinical manifestations. The biosafety risks caused by tissue biopsy samples from scrub typhus patients make it impossible to analyse these samples in BSL-2 laboratories [5].

NGS, a revolutionary development in first-generation sequencing methods, can simultaneously sequence hundreds of thousands to millions of DNA molecules with high throughput and short detection cycles [20]. mNGS can detect all pathogens in samples even when small amounts of pathogens are present [20]. Due to its high sensitivity, short detection cycle, and cost-effectiveness considerations, mNGS might be a potential diagnostic method that can partially replace traditional detection methods [20]. However, the use of NGS has rarely been reported in the case of scrub typhus presenting as a UTI. To the best of our knowledge, another case of *O. tsutsugamushi* infection detected by mNGS in a patient with atypical manifestations was reported by Wu et al. [21]. In our case report, *O. tsutsugamushi* was confirmed to be the pathogen on Day 13 of illness (20th March), and doxycycline treatment was demonstrated to be effective. In this case, the protocol of mNGS in our hospital refers to the information provided by Blauwkamp et al. [22]. mNGS detected trace pathogens in an accurate and rapid manner to save considerable time, thereby effectively avoiding the aggravation of the patient's condition and the chronic migration of the disease and even eliminating severe DIC. The results of mNGS directly affected the patient’s care, ultimately resulting in a satisfying outcome.

mNGS is likely a valuable method to diagnose scrub typhus, especially in complicated cases with atypical features. It is worth noting that the cost of mNGS, the practicality of using the method in underdeveloped areas with limited resources, potential limitations and obstacles in the clinical application of NGS, and the utility of NGS in overall clinical cases of scrub typhus warrant further research.

In summary, we used mNGS to diagnose a case of scrub typhus in a febrile patient without an eschar but with a UTI and high D-dimer levels. mNGS may be a useful method for identifying the pathogen responsible for infections without typical clinical symptoms. In the diagnosis of acute febrile patients with atypical clinical features, clinicians should consider atypical pathogens, such as *O. tsutsugamushi*, in patients with UTIs.

## Data Availability

All data generated or analysed during this study are included in this published article.

## Abbreviations

mNGS: Metagenomics next-generation sequencing
NGS: Next-generation sequencing
UTI: Urinary tract infection
WBC: White blood cell count
SAA: Serum amyloid A protein
CRP: C-reactive protein
PT/INR: Prothrombin time/International normalized ratio
ALT: Alanine aminotransferase
AST: Aspartate aminotransferase
NAATs: Nucleic acid amplification tests
DIC: Disseminated intravascular coagulation
